# Oxidative stress and inflammation as central mediators of atrial fibrillation in obesity and diabetes

**DOI:** 10.1186/s12933-017-0604-9

**Published:** 2017-09-29

**Authors:** Basil S. Karam, Alejandro Chavez-Moreno, Wonjoon Koh, Joseph G. Akar, Fadi G. Akar

**Affiliations:** 1 Cardiovascular Institute, Icahn School of Medicine at Mount Sinai, New York, NY USA; 2 Section of Cardiovascular Medicine, Yale University School of Medicine, New Haven, CT USA

**Keywords:** Atrial fibrillation, Diabetes, Oxidative stress, Electrical remodeling, Fibrosis

## Abstract

Atrial fibrillation (AF) is the most common sustained cardiac arrhythmia in humans. Several risk factors promote AF, among which diabetes mellitus has emerged as one of the most important. The growing recognition that obesity, diabetes and AF are closely intertwined disorders has spurred major interest in uncovering their mechanistic links. In this article we provide an update on the growing evidence linking oxidative stress and inflammation to adverse atrial structural and electrical remodeling that leads to the onset and maintenance of AF in the diabetic heart. We then discuss several therapeutic strategies to improve atrial excitability by targeting pathways that control oxidative stress and inflammation.

## Background

Atrial fibrillation (AF) is the most common sustained cardiac arrhythmia. Several risk factors promote AF, among which diabetes mellitus has emerged as one of the most important [1, 2]. A meta-analysis by Huxley et al. [3] revealed that diabetic patients exhibit ~ 40% greater risk of developing AF than their non-diabetic counterparts. In addition, obesity, a major component of the metabolic syndrome that promotes diabetes, is also independently associated with AF [2, 4, 5].

The growing recognition that obesity, diabetes and AF are closely intertwined epidemics has spurred major interest in uncovering their mechanistic links. In recent years, numerous lines of evidence have implicated oxidative stress and inflammation as central mediators of AF in metabolically-stressed hearts [6–8]. For one, mitochondria isolated from atrial tissues of diabetic patients [9] and animal models [10] exhibit increased emission of reactive oxygen species (ROS) due, in part, to impaired electron transport and hyperglycemia [11, 12]. Moreover glucose fluctuations which are common in diabetes promote ROS overproduction [13, 14]. The ensuing mitochondrial dysfunction and DNA damage are central to the progression of a host of cardiovascular diseases, including diabetic cardiomyopathy and AF. Other important sources of ROS that are altered in the diabetic heart include Xanthine oxidase, NADPH oxidase, Monoamine oxidase, Protein Kinase C, Nitric oxide synthase (NOS), and Advanced glycation end-products (AGE) [15]. In addition to increased ROS generation, antioxidant defense systems such as glutathione are depleted in the atria of diabetic hearts [11]. Mismatch between ROS scavenging and generation promotes oxidative stress and inflammation. In what follows, we review mechanisms by which these two key factors cause atrial structural and electrical remodeling in the diabetic heart. We then discuss several therapeutic approaches for diabetes-related AF, which have the common feature of ameliorating oxidative stress and reducing inflammation. This article is intended as an update on the evolving link between oxidative stress, inflammation, and AF in the diabetic heart [16]. For a more comprehensive viewpoint, we refer the reader to other excellent reviews on the subject matter [6, 17–20].

## Oxidative stress in diabetes and obesity exacerbates atrial structural remodeling

Structural remodeling (atrial enlargement, increased fat depots, interstitial fibrosis) is a major factor by which obesity and diabetes mellitus promote AF [21]. Clinically, pericardial fat volume which correlates with left atrial enlargement [22–24] has been associated with increased risk of AF [22, 25]. There is strong evidence, however, to suggest that this relationship is influenced by race. Specifically, in the Multi-Ethnic Study of Atherosclerosis and the Jackson Heart Study, Hispanics but not African Americans, Whites, or Chinese Americans exhibited significant association between pericardial fat and AF risk after adjusting for body-mass index [26]. Nonetheless, the pathophysiological significance of pericardial fat is illustrated by its ability to predict success of AF ablation procedures [25]. Finally, reversal of atrial fat by weight loss is associated with reduced AF burden and improved cardiac performance [27, 28].

Given its direct apposition to the myocardium, pericardial fat is an active mediator of cardiovascular pathology, including increased AF vulnerability [29–31]. Importantly, pericardial fat is a visceral adipose tissue that possesses endocrine and paracrine properties. As such, it expresses both anti-inflammatory adipokines (e.g. adiponectin, omentin, etc.) and pro-inflammatory cytokines (including interleukin (IL)-1β, IL-6, IL-8, TNF-α) that readily influence atrial excitability and structure [32]. In addition to serving as a pro-inflammatory source, direct infiltration of the atrial myocardium by adipocytes disrupts the myocardial architecture causing conduction slowing, a critical factor that promotes the maintenance of AF circuits [33].

Interstitial fibrosis is another prominent feature of atrial structural remodeling in diabetes. It involves the formation of collagen-rich myocardial tissue which also disrupts cell-to-cell coupling, hinders action potential propagation, and promotes reentrant excitation underlying fibrillatory activity. Moreover, fibroblast proliferation and differentiation into myofibroblasts results in adverse heterocellular (myocyte-myofibroblast) interactions through electrical, mechanical and biochemical coupling.

Finally, atrial stretch and enlargement in obese patients promote the onset and maintenance of AF by triggering stretch-activated channels and increasing the pathlength across which multiple reentrant circuits can form. In humans and large animal models, chronic AF requires a structurally remodeled atrial substrate which arises from a multitude of pro-inflammatory processes linked to oxidative stress. Here, we focus on three key pathways that are known for their relevance in obesity and diabetes.

### AGE–RAGE axis

Cross-talk between advanced glycation end products (AGE) and their receptors (RAGE) with the dipeptidyl peptidase-4 (DPP-4)-incretin system has been implicated in the pathogenesis of a number of diabetic complications, including retinopathy, nephropathy, and atherosclerosis [34, 35]. AGE levels are elevated in states of hyperglycemia and oxidative stress that arise in the diabetic heart [34]. Kato et al. [36] were among the first to highlight the putative role of the AGE–RAGE axis in diabetes-induced atrial fibrosis. Indeed, they reported markedly elevated levels of atrial fibrosis and RAGE expression in the atria of streptozotocin-induced diabetic rats, a standard model of type-1 diabetes mellitus [36]. Interestingly, fibrosis in this model was partially reversed following treatment with an AGE inhibitor suggesting a causal relationship between AGE levels, atrial fibrosis, and AF [36]. Raposeiras-Roubin et al. [37] reported increased expression of AGE and soluble RAGE in the venous blood of patients in permanent AF compared to those in sinus rhythm. More recently, Begieneman et al. compared AGE levels in the left atrial appendages (a hotspot for AF triggers) of AF patients compared to controls, and found increased N(ε)-(carboxymethyl)lysine which coincided with a marked rise in the number of inflammatory cells [38].

Although the exact mechanism by which the AGE–RAGE axis promotes atrial fibrosis remains unclear, it likely stems from the interaction of AGE with molecules in the basement membrane of the extracellular matrix. Binding of AGE to their receptors increases the expression of inflammatory mediators, namely NF-κB, which, in turn, causes tissue remodeling and damage [39]. Of note, AGE-mediated rise in NF-κB levels increases RAGE expression, causing further ROS elevation in a vicious cycle that exacerbates oxidative stress and inflammation, and therefore promotes disease progression.

### Transforming growth factor β1

TGF-β1, a member of the transforming growth factor superfamily of cytokines that mediates cell proliferation and differentiation, is also elevated in diabetes [40]. This, in turn, modulates inflammatory processes in multiple organs, including the heart. The relevance of this pathway to AF has been demonstrated by multiple groups [41–43]. For example, Liu et al. [44] demonstrated increased atrial interstitial fibrosis, atrial ion channel remodeling, and AF vulnerability that were accompanied by elevated TGF-β1 levels in alloxan-induced diabetic rabbits. Targeted gene-based reduction of TGF-β1 signaling via constitutive expression of dominant negative TGF-β type II receptor in the posterior left atrium decreased the extent of replacement fibrosis, and in doing so, improved atrial conduction and decreased AF propensity [43]. A comprehensive analysis of the differential regulation of atrial versus ventricular remodeling by TGF-β signaling is required in future studies.

### RhoA–ROCK pathway

Another pathway that promotes atrial fibrosis is mediated by the Ras homolog gene family member A (RhoA), a GTPase protein that regulates cell adhesion, smooth muscle contraction, and reorganization of the actin cytoskeleton [45]. The so-called RhoA/ROCK pathway was initially implicated in ventricular fibrosis in a model of type-1 diabetes mellitus [46]. More recently, Chen et al. [47] extended this observation to the atria of diabetic animals. Specifically, in a rat model of type-2 diabetes mellitus that exhibits significant atrial fibrosis, they found increased expression levels of RhoA and its effectors (ROCK1 and ROCK2) at both the mRNA and protein levels. Treatment of these animals with the Rho-kinase inhibitor fasudil hydrochloride hydrate suppressed the pro-fibrotic program by decreasing the expression of RhoA and its effectors [47].

## Oxidative stress in diabetes exacerbates atrial electrical remodeling

Atrial electrical remodeling is a cause and consequence of AF. At the tissue level, electrical remodeling comprises effective refractory period shortening, conduction velocity slowing, wavelength reduction, and frequent atrial ectopy caused by calcium (Ca^2+^)-dependent triggered activity [48–50]. While Ca^2+^-mediated triggers serve as the critical initiators of AF, an appropriate substrate formed through progressive electrical and structural remodeling of the atria is required for the long-term perpetuation of the arrhythmia and its conversion from paroxysmal to chronic forms.

At the molecular level, electrical remodeling arises from altered expression and function of a host of ion channel, Ca^2+^ cycling, and gap junction proteins. While a comprehensive discussion of mechanisms by which these proteins are regulated by redox signaling is outside the scope of this review article, we focus on three key players, namely the ryanodine receptors (RYR2), sodium (Na) channels, and gap junction proteins owing to their importance to AF initiation and maintenance. In what follows, we highlight key studies linking oxidative stress to the malfunction of these critical targets.

### Oxidative stress in diabetes promotes Ca^2+^-mediated triggers and the initiation of AF

Triggered activity caused by delayed afterdepolarizations (DADs) is typically required for the initiation of AF. DADs arise from increased Ca^2+^ leak from the sarcoplasmic reticulum (SR) via RYR2. This, in turn, causes diastolic SR Ca^2+^ release events, which activate the Na^+^/Ca^2+^ exchanger (NCX), producing a transient-inward current that causes membrane depolarization. If large enough, these depolarizations trigger propagated wavefronts forming premature atrial beats that can initiate AF. Numerous studies have implicated hyperphosphorylation of RYR2 via calmodulin-dependent protein kinase II (CaMKII) in arrhythmogenic SR Ca^2+^ leak in ventricular and atrial myocytes of diseased hearts. CAMKII, which typically requires the binding of Ca^2+^ to calmodulin for its activation, is also triggered by oxidation. Multiple groups have highlighted the role of mitochondria-derived ROS in the oxidation and therefore activation of CAMKII leading to the arrhythmogenic hyperphosphorylation of RYR2. Indeed, mitochondria-derived ROS have been shown to cause AF in multiple transgenic mouse models harboring leaky RYR2 channels [51]. With regards to diabetes, Joseph et al. [52] demonstrated that cardiac lipid overload secondary to peroxisome proliferator-activated receptor gamma (PPAR-γ) overexpression was associated with mitochondrial oxidative stress and increased SR Ca^2+^ leak via oxidized RyR2 channels. These defects likely resulted in frequent ventricular ectopy, which was reversed by treatment with a mitochondria targeted anti-oxidant [52]. Whether a similar mechanism promotes atrial ectopy and AF in diabetes awaits further study.

### Abnormal atrial conduction and the maintenance of AF

In large animals and humans, AF is maintained by reentrant excitation forming stable or meandering rotors, leading circle reentry, or multiple circulating wavelets. Unidirectional conduction block is a prerequisite for reentrant excitation and conduction slowing is a key predisposing factor for conduction block. Conduction slowing causes wavelength shortening, which in turn, promotes the stability of AF circuits. Studies by multiple groups have demonstrated substantial conduction slowing and reduced conduction reserve in the atria of diabetic animals. In general, these changes arise as a consequence of structural remodeling (covered in the previous section), decreased Na channel activity, or altered expression, phosphorylation, and localization of gap junction proteins.

Atrial gap junctions are formed by the assembly of connexin (Cx) proteins, namely Cx40 and Cx43. Downregulation of Cx40 as well as hyperphosphorylation and downregulation of Cx43 have been implicated in the electrical remodeling of the diabetic heart that culminates in conduction slowing and AF [53]. However, these molecular changes have not been corroborated in all studies. For example, Mitasikova et al. [54] demonstrated paradoxical upregulation (not downregulation) of Cx43 with a decrease (not increase) in Cx43 phosphorylation. These seemingly conflicting results may be attributed to the specific sites of phosphorylation on Cx43 or the stage of diabetes. As case in point, we reported stage-dependent discrepancies in Cx43 phosphorylation in ventricular myocardium of rats with pressure overload hypertrophy [55]. Specifically, we found hyperphosphorylation and increased expression of Cx43 at the early (compensated) stage of hypertrophy that were followed by marked downregulation and dephosphorylation of the protein at late stages of remodeling [55]. Notably, conduction slowing was observed at both early and late stages of remodeling but was more severe during the latter [55]. While the molecular mechanisms underlying gap junction remodeling leading to AF are largely unknown, there is substantial evidence that oxidative stress plays a major role. For one, oxidative stress alters the atrial expression of Cx40 and Cx43 as well as the size of atrial gap junctions in a model of intermittent hypoxia mimicking obstructive sleep apnea [56]. Moreover, oxidative modification of tyrosine-mediated signaling plays a key role in Cx43 remodeling during the progression of streptozotocin-induced diabetes [57]. Finally, oxidative stress disrupts Cx43 forward trafficking to the intercalated disk resulting in abnormal gap junction coupling [58].

Na channel activity plays a major role in mediating proper action potential conduction across the heart. In alloxan-induced diabetic rabbits that are prone to AF, Liu et al. [44] demonstrated decreased I_Na_ density that was likely caused by the pro-inflammatory rise in NF-κB levels. In addition to its regulation by inflammatory cytokines, I_Na_ in ventricular myocytes is highly sensitive to oxidative stress, elevated NADH levels and protein kinase C activation [59]. Remarkably, treatment with a mitochondria-targeted antioxidant reversed this defect in murine models and in ventricular samples from patients with non-ischemic heart failure [59]. Although the role of NADPH-ROS signaling in the modulation of atrial Na channel expression and gating will require direct investigation in models of diabetes, elegant findings by the Dudley group highlight the importance of metabolic pathways in the regulation of impulse formation and conduction via direct effects on I_Na_ activity [60].

A master metabolic pathway which is highly relevant in the setting of diabetes is mediated by liver kinase B1 (LKB1), an upstream kinase with multiple downstream effectors. One of those effectors is 5′ adenosine monophosphate-activated protein kinase (AMPK), a critical component of the metabolic stress response of the heart to injury and a target of the anti-diabetic agent Metformin. The relevance of this metabolic pathway in diabetes is underscored by studies demonstrating the cardioprotective efficacy of AMPK activation in Goto Kakizaki type-2 diabetic rats [61] and streptozotocin-induced type-1 diabetic mice [62]. While LKB1 knockdown was shown by multiple groups to cause adverse ventricular remodeling, hypertrophy and AF, the underlying mechanisms by which defective LKB1 signaling promotes atrial arrhythmias remained unclear. Specifically, whether loss of LKB1 *per se* causes primary atrial electrical remodeling and AF independently of ventricular dysfunction and heart failure remained unknown. Using LKB1 knockout mice, we showed early remodeling of atrial gap junctions and ion channels that preceded ventricular remodeling or the spontaneous onset of permanent AF [63]. Specifically, knockdown of LKB1 led to significant downregulation of atrial Cx40 and I_Na_ peak density, causing prolonged intra-atrial depolarization and inter-atrial conduction block [63]. Future studies aimed at investigating the role of this metabolic pathway to diabetes-related AF are needed.

## Therapies targeting oxidative stress and Inflammation for treatment of AF in diabetes

Standard pharmacotherapies targeting sarcolemmal ion channels for AF treatment or prevention are associated with limited efficacy and potential toxicity, including risk of pro-arrhythmia. This is largely due to the fact that ion channel ligands typically modulate the activities of atrial and ventricular ion channels alike. As a result, they often disrupt ventricular electrophysiological properties, especially in the context of a chronic disease such as diabetes that causes adverse ventricular remodeling and fibrosis. In light of this major challenge, there is growing interest in developing non-ion channel targeting agents that have the potential to alter the underlying atrial substrate without provoking pro-arrhythmic effects [64–68]. Since oxidative stress and inflammation are critical upstream mediators of adverse atrial structural and electrical remodeling, targeting pro-oxidant and pro-inflammatory factors may hold substantial promise for anti-AF therapies. In what follows, we provide several notable examples. This, however, is by no means intended as a comprehensive listing of promising therapies.

### Pioglitazone

Thiazolidinediones (TZDs) are a class of PPAR-γ activators that exhibit potent glucose lowering efficacy, and hence are widely used in patients with insulin resistance and diabetes mellitus. In addition, TZDs which exert a number of pleiotropic effects including decreased inflammation and adiposity, are attractive agents for chronic cardiovascular disorders. A recent meta-analysis by Zhang et al. [69] highlighted the potential for TZDs in conferring protection against AF in patients with diabetes mellitus. Experimentally, pioglitazone, a prominent member of this class of agents, has been shown to inhibit AF by modulating pro-inflammatory and hypertrophic signaling pathways via suppression of TGF-β1, tumor necrosis factor alpha (TNF-α), and phospho-ERK levels [70–73]. Moreover, administration of pioglitazone results in the depolarization of the inner mitochondrial membrane with a reduction in maximal ROS production [74]. The dual antioxidant and anti-inflammatory effects of pioglitazone are therefore thought to ameliorate atrial electrical and structural remodeling [19, 70, 75–77]. PPAR-γ agonists, including pioglitazone, inhibit inducible NOS (iNOS) activity, enhance endothelial NOS bioavailability and reduce NADPH oxidase-dependent superoxide production [19]. Finally, pioglitazone increases soluble RAGE levels while decreasing overall RAGE expression, effects that are consistent with improved structural remodeling and anti-fibrotic action [75, 78, 79]. In light of these encouraging findings, more studies are needed to determine the electrophysiological effects of pioglitazone, and its efficacy in the prevention and treatment of AF in animal models of diabetes.

### Polyunsaturated and nitrated fatty acids

Omega-3 polyunsaturated fatty acids (PUFAs) provide beneficial effects in insulin resistance and type-2 diabetes mellitus by enhancing anti-oxidant defense mechanisms. They do so by reducing the accumulation of fatty acid metabolites, providing cytoprotection for pancreatic β-cells, decreasing inhibitor of NF-κB and c-Jun N-terminal kinase (JNK) pathways, activating AMPK stress response signaling, and modulating PPAR-γ activity [80]. With regards to AF, supplementation with PUFAs and antioxidant vitamins decreases NF-κB activation likely due to the attenuation of the inflammatory and pro-oxidant state [81].

Increasing lines of evidence support the notion that PUFA supplementation is cardioprotective and likely to exert anti-arrhythmic effects in the setting of diabetes [82–85]. Acute administration of eicosapentaenoic acid (EPA) inhibits the formation of noradrenalin-induced DADs and triggered activity [86, 87]. In addition, EPA reduces pulmonary vein firing, an established AF driver, through NO-dependent mechanoelectrical feedback [87]. Finally, Rudolph et al. [88] demonstrated a potent protective effect of nitrated fatty acids against angiotensin II mediated atrial fibrosis and AF. This protection was mediated by suppressing Smad2-dependent myofibroblast differentiation and xanthine oxidase-dependent atrial superoxide levels [88]. Further studies are needed to comprehensively define the electrophysiological effects of fatty acids on atrial excitability and ion channel function in models of diabetes.

### Vitamins and anti-oxidants

Since oxidative stress plays a critical role in the pathogenesis of AF, the use of vitamins C and E holds promise as an adjunctive therapeutic strategy. Besides their ROS scavenging capacity, these vitamins exert other modulatory actions including downregulation of NADPH oxidase and up-regulation of endothelial NOS activities. This, in turn, increases NO synthesis, decreases ROS formation and improves overall vascular tone [89]. Studies testing the anti-AF effects of vitamin C have suggested modest benefits [90–94]. Although Carnes et al. [95] found a substantial reduction in the incidence of post-operative AF in patients supplemented with Ascorbate, double-blind, placebo-controlled multi-center clinical trials based on these early studies ultimately showed mixed results [96–98]. Development of targeted anti-oxidant approaches that interfere selectively at the level of the defective protein or organelle are likely to produce more favorable outcomes.

One such approach is the use of mitochondria-targeted coenzyme Q (MitoQ), an antioxidant enzyme acting specifically on mitochondria to ameliorate ROS-induced injury. Administration of MitoQ to rodents was found to be safe [99] and effective against oxidative stress caused by ischemia–reperfusion (IR) injury [100], hypertension [101], and kidney damage in type-1 diabetes mellitus [100, 102, 103]. MitoQ also elicited protective effects against doxorubicin-induced cardiotoxicity [104]. Findings by Escribano-Lopez et al. [105] support the notion that the dual antioxidant and anti-inflammatory action of MitoQ is mediated, in large part, by decreased ROS production in the leukocytes of type-2 diabetic patients. In recognition of the ability of MitoQ to improve oxidative stress and inflammation, human trials have been undertaken, primarily for Parkinson’s disease [106] and chronic hepatitis C [107]. Testing of mitochondria-targeted antioxidants in patients with diabetes mellitus is warranted considering the disappointing outcome of trials examining the efficacy of vitamins as vehicles for anti-oxidant treatment [105].

### Statins

Antioxidant and anti-inflammatory effects of statins have been extensively described in animal models and humans. Statins (such as Atorvastatin) reduce the risk of myocardial infarction, stroke, and death, by primarily inhibiting ROS levels and inflammation [108]. Underlying mechanisms include enhancement of eNOS activity and NO bioavailability. This, in turn, inhibits the overexpression of adhesion molecules and preserves mitochondrial membrane potential in response to oxidative stress [109, 110].

At the cellular level, Atorvastatin inhibits angiotensin-mediated cell injury by suppressing ROS production in neonatal rat ventricular myocytes [111]. More importantly, Atorvastatin treatment prevents atrial electrical remodeling, including effective refractory period shortening and reduction of the L-type calcium current in a rabbit model of tachycardia-pacing induced AF [112]. Clinically, statins confer their greatest benefit in the prevention of post-operative AF [94, 113, 114]. On the other hand, statins seem to offer limited benefits in the primary prevention of AF. Further studies are needed to determine the efficacy of statins in the prevention or management of obesity and diabetes related AF.

### Dipeptidyl peptidase inhibitors

Emerging evidence supports a role for DPP-4 inhibitors in the treatment of AF. DPP-4 is a transmembrane glycoprotein that has two key substrates, namely glucagon like peptide-1(GLP-1) and glucose-dependent insulinotropic peptide (GIP) [115]. The mechanism by which DPP-4 modulates glucose metabolism involves the inhibition of GLP-1 degradation and glucagon secretion, as well as enhancement of beta-cell function, which stimulates insulin secretion [116, 117].

DPP-4 inhibition mediates anti-inflammatory, antioxidant, and anti-fibrotic effects which are critical for the prevention of AF in diabetes and obesity [118–120]. Zhang et al. [10] found that Alogliptin, a DPP-4 inhibitor, improved atrial remodeling by decreasing mitochondrial ROS, preventing mitochondrial membrane depolarization, enhancing mitochondrial biogenesis, and alleviating mitochondrial swelling in diabetic rabbits. The safety of this approach was highlighted by Wu et al. [121] who found that multiple DPP-4 inhibitors (alogliptin, linagliptin, saxaglipton, sitagliptin, teneligliptin, and vildagliptin) were associated with less adverse gastrointestinal side-effects compared to GLP-1 receptor agonists, metformin, and α-glucosidase inhibitors.

## Summary

Atrial fibrillation, obesity, and diabetes mellitus are intertwined disorders that are linked through oxidative stress and inflammation. Both factors exacerbate atrial electrical and structural remodeling leading to the formation of an adverse substrate that facilitates AF initiation and maintenance. Developing mechanism-based strategies targeting oxidative stress and inflammation will likely generate new, safe, and effective therapeutic opportunities for combatting the growing epidemic of diabetes-related AF.
